# Person-centred care during treatment with nasal esketamine — a qualitative study

**DOI:** 10.1186/s12912-025-02943-y

**Published:** 2025-03-14

**Authors:** Lina Klysing, Ingrid Larsson, Katrin Häggström Westberg

**Affiliations:** 1 School of Health and Welfare, Halmstad University, Halmstad, Sweden; 2 Affecta Psychiatric Clinic, Halmstad, Sweden

**Keywords:** Nasal esketamine, Person-centred care, Treatment-resistant depression, Qualitative

## Abstract

**Background:**

Patients suffering from treatment-resistant depression may be treated with nasal esketamine. The treatment requires administration in a healthcare facility and monitoring by a nurse. Existing research has mainly focused on the clinical effect of nasal esketamine, while less is known about patients’ experiences of treatment. A person-centred framework provides valuable insights into care and treatment. By using person-centredeness, the patient turns into a respected contributor in the delivery of care, enabling improved outcomes, better use of resources, reduced costs and increased satisfaction with care. Thus, the aim was to explore patients’ experiences of treatment with nasal esketamine utilizing the person-centred nursing framework by McCormack and McCance.

**Methods:**

The study had a qualitative design with an abductive approach. Twenty patients with treatment-resistant depression who had received at least eight treatments of nasal esketamine were interviewed. A thematic analysis was conducted, based on the four constructs of PCC: prerequisites, care environment, person-centred process and expected outcomes. The analysis generated 11 subthemes, reflecting the contextuality of the PCC nursing framework.

**Results:**

The results highlighted the importance of working in a person-centred manner to achieve increased patient satisfaction and treatment outcomes. Patients highlighted the importance of relationships with competent and engaged nurses and a supportive care environment for treatment outcomes. The physical care environment influenced patients’ well-being, and organizational aspects, such as continuity of staff, flexibility, and being allowed a private space, were also crucial during esketamine treatment. These aspects fostered feelings of security and contributed to achieving the desired outcome.

**Conclusion:**

This study identified that key aspects of PCC; competent nurses and respect for the patient, comfort and personalization of the care environment, support and involvement are important in ensuring patient satisfaction, quality and ultimately the desired treatment outcomes in esketamine treatment. By adopting a person-centred approach, nurses can increase patient well-being and the quality of care in nasal esketamine treatment.

**Clinical trial number:**

Not applicable.

**Supplementary Information:**

The online version contains supplementary material available at 10.1186/s12912-025-02943-y.

## Background

Depression is associated with higher mortality rates and affects well-being and quality of life in the same way as a major physical illness [1]. There are also major consequences of depression for the family and society, through increased healthcare costs and reduced productivity [2]. As approximately 30% of people with depression are considered treatment-resistant (TRD), in that they do not respond to currently available medications [3], the need for new and improved treatments is urgent. Nasal esketamine (ESK) (Spravato©) is a novel, fast-acting anti-depressant treatment for patients with TRD, approved in the European Union since 2019 [4]. ESK was originally developed from ketamine, traditionally used as a dissociative anaesthetic, and has a clinically proven positive effect on depressive symptoms [5–7]. It is an S-enantiomer of ketamine, targeting the glutamatergic system. The mode of action is through the non-competitive, non-selective antagonist effect on NMDA receptors, thought to increase neurotrophic signalling and dopaminergic transmission [8]. It is considered to be an efficacious and fast-acting anti-depressant treatment for patients with TRD, with a distinct safety profile and a different, intranasal method of administration [4, 8]. According to approved guidelines, ESK must be administered concomitantly with an SSRI/SNRI, prescribed by the treating psychiatrist after clinical judgment [4]. The induction phase is usually 4 weeks, with treatment initiating at 56 mg esketamine at the starting day, increased to 84 mg at subsequent visits. The first four weeks, treatment is administered twice weekly. Evidence of therapeutic benefit is evaluated after four weeks. If the patient shows signs of clinical improvement, treatment is continued weekly or bi-weekly, with the possibility of increasing dosing frequency should the patient relapse. Treatment is recommended to continue for at least six months after depressive symptoms improve.

Common side effects of ESK are visual and auditory perceptual changes, altered proprioception, sedation, disassociation, and occasionally ‘mystical’ experiences. Dizziness, nausea, anxiety, lethargy, hypoesthesia (loss of sensation), blood pressure elevation, vomiting, and feeling intoxicated are also common, but most side effects disappear within 90 min of administration [4, 7, 9]. ESK is administered in a healthcare facility and the patient is monitored for up to three hours after administration [7]. Thus, patients receiving ESK spend a substantial amount of time at the healthcare facility in the presence of a nurse, while being in a vulnerable state of mind, both related to the depression itself, but also because of the impact of the medication. The expert consensus statement on the practical recommendations for the management of TRD with ESK stresses that patient education is vital for compliance and adherence, as is the management of patient expectations of potential adverse events [9], but it seems only a limited number of studies have explored the patient experience of ESK [10].

Person-centred research shows a need to improve existing treatment options, by prioritizing patient well-being and collaboration with healthcare professionals, which is also the basis of the person-centred framework (PCC) [11, 12]. PCC is described as a mid-range healthcare theory based on evaluations of patient-centred care approaches to nursing and the patient’s values, needs, choices, and preferences in care planning and delivery [13]. PCC contains four constructs: prerequisites, care environment, person-centred processes, and outcomes. The goal of PCC is to reduce patient vulnerability through therapeutic engagement and to establish a collaborative care relationship between the patient and nurse, where both have equal power in decision making. The nurse-patient relationship is built on mutual trust, understanding, and knowledge sharing [13]. Prerequisites focus on the attributes of the nurse; being professionally competent, committed and being clear on personal beliefs and values. The care environment encompasses not only the organizational aspects of a smoothly functioning team, but also a flexible and supportive organization and the physical environment. Person-centred processes focus on delivering holistic care, with respect to the patient as a whole being, engaging in authentic, empathic and shared decision-making. The expected outcome reflects evaluation by a patient of a good care experience [14]. This framework of care has been shown to contribute to better outcomes for both patients and organizations, in the form of increased satisfaction with care and reduced costs [15, 16]. PCC also improves communication and the relationship between the nurse and the patient, which has a positive effect on adherence to treatment for depression, leading to better treatment outcomes [17–19].

In ESK treatment, it is crucial to optimize outcomes, as these patients have a history of not responding to previous treatments. During treatment sessions, it is important to prioritize patients’ well-being, because they may have altered sensory experiences and dissociative side effects, which make them particularly vulnerable. This study contributes to increased knowledge of patients’ experiences of ESK treatment, through the lens of PCC.

## Methods

The aim was to explore patients’ experiences of treatment with nasal esketamine utilizing the person-centred nursing framework by McCormack and McCance.

### Study design and setting

This study had a qualitative design and employed semi-structured interviews, focusing on capturing and describing the experiences of patients receiving ESK treatment for TRD at a psychiatric out-patient clinic in Sweden. As ESK treatment is administered by a registered nurse, in this study, the term “nurse” signifies a registered nurse.

### Participants and recruitment

A consecutive recruitment strategy was employed. All patients eligible for inclusion had been diagnosed with TRD by a psychiatrist and had received at least eight treatments of nasal esketamine, the minimum standard of treatment for induction and evaluation. They had no known aneurysmal vascular disease, hypertension or cardiovascular incident within the six weeks preceding the treatment start. The patient’s blood pressure had been controlled before initiation of the treatment. Information letters and consent forms with pre-paid envelopes were sent to 32 patients with TRD treated with ESK. After one week, patients were contacted by non-treating staff from the out-patient clinic via telephone and allowed to ask questions. In all, 22 patients agreed and provided written consent. The researcher contacted the patients who agreed to participate in the study by email or text message, to schedule an interview. Two patients who provided consent did not reply to messages or emails, resulting in 20 interviews conducted, with 11 women (55%) and 9 men (45%). The patients were aged 19–72, with an average age of 46. Fifteen patients had studied at university, four at secondary school, and one at primary school. Professions ranged across diverse fields, such as teaching, IT, and engineering. Sixteen patients were born in Sweden, while the remaining four were born abroad. Eleven lived with a partner, while nine lived alone or with their parents. The duration of the depression varied from between 1.5 and 42 years.

### Data collection

The data were collected by way of individual, semi-structured interviews – a suitable method for exploring people’s thoughts and opinions. Such interviews involve the interviewer writing a guide with relevant questions about the subject under investigation, while allowing patients to express themselves in their own words, in order to provide a comprehensive account. An interview guide was developed by the research team, pilot-tested and subsequently used for all the interviews (see supplementary file A). The interviews were conducted between November 2023 and February 2024. LK conducted nineteen interviews, and one was done by KHW. Five interviews were held face-to-face at the psychiatric clinic, four via phone, and 11 interviews were done digitally, using ZOOM or Teams. Interview length ranged from 29 to 89 min (Median = 48), with a total interview length of 15 h and 9 min. All interviews were digitally recorded and transcribed verbatim.

### Data analysis

The collected data was analysed using thematic analysis [20], with an abductive approach [21]. The analysis was done in steps (see Table 1), beginning with familiarization of the material, and open and inductive coding, performed by author LK. The codes were sorted into the various PCC constructs and compared within each construct. Sub-themes were identified and arranged on the basis of McCormack and McCance’s four constructs: prerequisites, care environment, person-centred processes, and expected outcomes [13]. The analysis was done in AtlasTi © (2002–2024 – ATLAS.ti Scientific Software Development GmbH, v8.7.1-2024-09-30). Coding was performed by one member of the research team (LK), with analysis and close collaboration between the research team. Two members of the research team were mental health credentialled nurses with several years of experience, whereas the third member had clinical experience primarily from the area of rheumatology, but extensive experience in methodological issues and nursing research within mental health. The background and expertise of the research team enhance the trustworthiness of this study.

**Table 1:** The analysis process

The aim was to explore patient experiences of treatment with nasal esketamine, utilizing a person-centred nursing framework	Step	Analysis method	Result
	A	Inductive analysis	69 meaning units were identified and were sorted into 17 codes
	B	Deductive analysis	Codes were sorted within the four constructs of PCC: prerequisites, care environment, person-centred processes, and expected outcomes
	C	Inductive analysis	Codes within the constructs were compared and 11 subthemes emerged
	D	Deductive analysis	The subthemes were sorted within the four constructs of PCC: prerequisites, care environment, person-centred processes, and expected outcomes

## Results

**Fig. 1:**
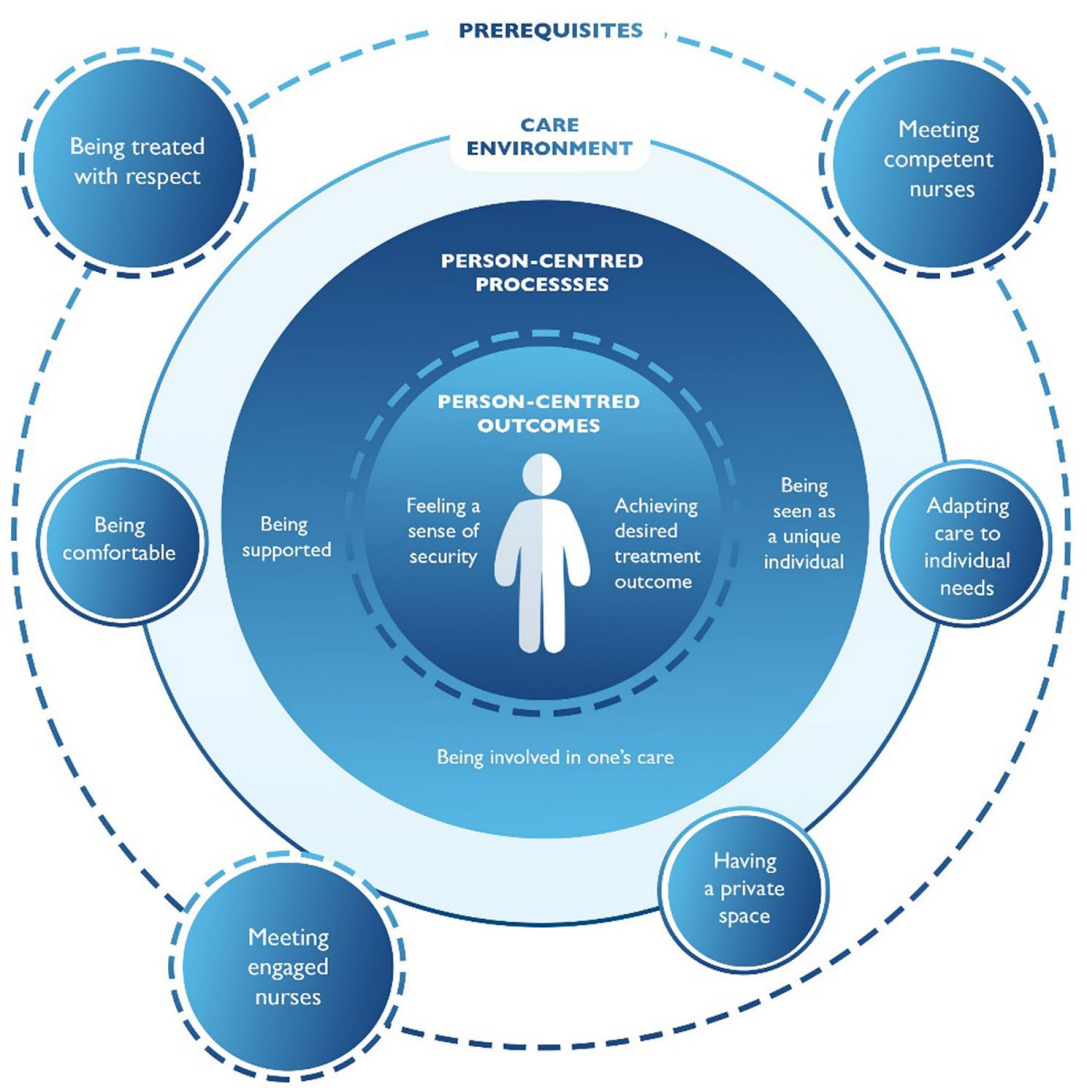
Overview of themes and subthemes depicting patients’ experiences of treatment with nasal esketamine, based on McCormack and McCance's nursing framework of person-centred care

Through the patients’ narratives of treatment with ESK, the four constructs of PCC were identified: the prerequisites entailed receiving care from competent and engaged nurses who treated the patients with respect; the care environment needed to be comfortable, providing patients a private space, and the care had to adapt to individual needs; person-centred processes required that patients were made to feel as unique individuals, supported, and involved in their own care. The expected outcomes were feeling a sense of security and achieving the intended treatment outcomes (see Fig. 1).

### Prerequisites

During ESK treatment, prerequisites for PCC were described in terms of the importance of meeting nurses who were competent and engaged. Patients also stressed the importance of being treated with respect.

#### Meeting competent nurses

Patients reported feeling protected and secure when they met competent nurses who could provide information and answer questions. They also valued feeling confident that nurses were doing what they could to meet their needs. *“Yes, they are professionals. You can ask questions; they have a lot of experience. They know about psychiatry. And then they are just very warm people who listen and take you in and follow you, move on in the process as if you are not just a patient. You feel … you almost have a bit of a family feeling, in a way, that I have come in here and I immediately felt that these people will help me and take care of me. And that is extremely important in psychiatry. Especially when, after so many years, you are completely abandoned, and you get this kind of treatment”.* (Participant no. 1)

#### Meeting engaged nurses

Patients described meeting engaged nurses who showed empathy, concern for the individual’s well-being, and a willingness to help make them feel secure and cared for. They also highlighted the value of nurses taking their time, showing patience, and demonstrating compassion in their care.*“Yes, they do their best. There is a genuine desire to help. So it’s a good atmosphere.”* (Participant no. 2).

#### Being treated with respect

To be treated with respect was a prerequisite for PCC. Patients felt seen and respected when they were listened to and taken seriously. Patients noted that a friendly reception and being greeted by name made them feel welcome and well-cared for, and that nurses with a calm, friendly, and gentle demeanour positively impacted their well-being.

*“Yes, NN has a very pleasant manner. I liked her as a person and so it was already nice there, so she didn’t do anything special. She was herself or a nurse or whatever. And the same applies … well, if her name is NN or NN (name of nurse). I can’t remember anyone else who was there and helped in a soft, nice way.”* (Participant no. 3).

### Care environment

During treatment with ESK, patients described the importance of the care environment in PCC feeling comfortable, having a private space, and adapting care to individual needs.

#### Being comfortable

It was essential for the patients to be comfortable during the treatment. Reclining armchairs were used and most patients were satisfied with these; however, some wished they had the option to lie down on a bed. Some patients felt cold during treatment and appreciated receiving a blanket. Patients appreciated the homely environment at the clinic, as opposed to a traditional, sterile hospital environment. Patients also highlighted that they appreciated the presence of a therapy dog at the clinic, as it provided a sense of calm and did not demand that the patient be happy.

*“I love animals, so that … But she’s so … When she’s in the same room, it’s like a calming effect on you, that there’s someone else coming to greet you and welcome you. It doesn’t matter if you’re crying or you’re looking a bit rubbish today, because you might not be at your best. So it doesn’t matter to her, because she always comes with her tail wagging. And then you make an effort, because it’s not her fault that I might not be at my best.”* (Participant no. 4).

During treatment, some patients reported heightened senses, with the room’s sound and light significantly affecting their well-being. Patients noted an increase in their hearing sensitivity, with some preferring complete silence in the room, while others preferred to hear music playing. The music varied, but some patients found that specific types of music eased their experience and made their experience more comfortable. Patients preferred the lighting to be dimmed or turned off, although some patients were okay with daylight. The importance of being informed about their options and having their sound and light preferences addressed before treatment was emphasized.

*“But an unreal experience, and the colours were enhanced, the curtains were not quite in place. My body was different, it was light and … a little dream-like state, and sounds were amplified. So I wanted to sit in a dark room, that was very important. No lights were allowed to be on.”* (Participant no. 5).

#### Having a private space

Patients highlighted the significance of having a calm and private environment during treatment. They emphasized that it was crucial not to share treatment rooms with other patients, as they felt vulnerable and in need of solitude while under the influence of ESK. Some patients found it comforting to receive their treatment in the same room every time, while this did not matter for others.

*“Do you think it’s important to come to the same room, or is it just as good to be in different ones? RESPONDENT: No, not if the whole environment, and I’m talking about the reception centre and the nurses there, was as safe as it was. It is possible that for some people, for some reason, they feel more secure in a certain room, but that was not the case for me; I felt secure in the premises in general and with the nurses.”* (Participant no. 6).

#### Adapting care to individual needs

Patients emphasized that organizational factors, such as continuity of nurses with knowledge of each individual and flexible appointment times were important. Patients had disparate needs for supervision during their stay in the clinic after receiving ESK. Some always wanted the nurse to be present, while others wanted privacy. Some wanted varying levels of supervision from one time to the next. This study showed that supervision was adapted to the patient’s wishes and needs, rather than following a routine, which the patients appreciated and found positive for their experience.

*“They usually asked do you want me to come in when you’re taking the next one, do you want me to stay, and stuff like that.”* (Participant no. 7).

### Person-centred processes

Patients receiving ESK emphasized the person-centred process in PCC with nurses supporting individuals, valuing their unique identity and involving them in decision making.

#### Being seen as a unique individual

Meeting the same nurse and building mutual trust was important for well-being and recovery. Patients also emphasized the importance of being seen as an individual by the nurse, rather than just a diagnosis or a number. This was done by being greeted by name and seeing that the nurse was interested in them. It was significant for their well-being to be heard and to be able to have conversations about their life in general, not just their mental health or treatment.

*“Yes, but it has been great. Really, and my nurse, that’s probably the best thing that could happen I think, that I got her. So, it was a great relationship. And then you talk about all sorts of things. You get to know each other perhaps on a more superficial level, but still, it was cosy to have one and the same [nurse] and feel that she was always there.”* (Participant no. 8).

#### Being supported

Patients described that the support of the nurse, which included both physical touch and comforting words, made it easier to cope with the side effects of ESK. The feeling of being supported by the nurse was facilitated by the fact that there was a reciprocal knowledge about one another and a progressive build-up of trust. As a result, patients did not always have to communicate their needs explicitly, as they were sometimes implicitly understood. Patients also expressed a feeling that the nurses were available and confident that they were there to help when needed. Some expressed that the support and relationship with the nurse felt as healing and important as ESK.

*“I understand that there are people who drop out of treatment, because it’s very intense, and in … but in the right circumstances,… and I really felt that I had such a nice support, a good support. So that I felt I could… I could cope when it came… when it was very overwhelming.”* (Participant no. 9).

“*A treatment that was vital for my life. And when I mean treatment, it’s … The esketamine in itself, well it has helped me a lot, but I don’t think I would have managed if I hadn’t had the support I have felt from the clinic.* “ (Participant no. 11).

#### Being involved in one´s own care

Patients reported that they had the opportunity to be involved in decisions regarding their treatment and the arrangement of the room. This was achieved by the nurse asking questions such as “What do you think would be best for you?” Patients felt that their opinions were respected and considered. When patients were unable or unwilling to participate in care decisions, nurses sensitively took on a greater role in decision making. Patients also stated that they received enough information about the options, advantages, and disadvantages, so that they could make informed decisions. Being involved in the decision-making process was important for patients to feel secure and in control. The times patients were not allowed to be involved in the decision-making process, or their wishes were not granted had to do with factors related to patient safety, such as receiving treatment under supervision and not at home or receiving a dosage that was not in line with recommendations. Patients expressed the importance of nurses being aware and respectful, even when they were unable to partake in decisions. In some cases, the nurse made decisions on their behalf, but patients expressed a trust that nurses were acting in their best interest.

*“It is probably as big as it can be without me taking over their role, that I can be involved and think and think and … They listen and if I am wrong, they explain. They don’t just say “No, but you don’t know this”, but they assume that I’m interested in it because it affects me and that I’ve read up on it and that … They listen and respect … Even when I’m wrong, they respect me. I guess that’s it, that I can have an influence on it. And it also feels more secure than if they just say “You’re going to try this for a month” and then you don’t know what it is at all.”* (Participant no. 10).

### Expected outcomes

Expected outcomes in PCC meant patients wanted to feel cared for and achieve their treatment goals.

#### Feeling a sense of security

Patients emphasized the importance of feeling safe and secure, to feel satisfied with their care experience. They had had previous experiences of not receiving adequate help, so it was important for them to feel cared for, listened to, and reassured that the nurse had the right skills and were doing what they could to help them. Patients reported a sense of reassurance when they found nurses to be available and attentive to their concerns.

*“There and then I was taken care of by competent medical healthcare professionals. Doctors and nurses. I didn’t think I was competent to interfere with what they were doing, but I described what I was experiencing, and I was confident that they were doing the right thing.”* (Participant no. 6).

#### Achieving desired treatment outcome

Patients in this study experienced a positive effect from treatment with ESK, feeling a sense of relief, improved coping with daily life, and an improved quality of life. The good relationship with the nurse and the care received during the treatment of ESK also positively impacted patients’ well-being. Some patients reported that it had a life-changing effect. Patients found it difficult to determine whether it was the medication or the nurse that made them feel better.

*“Maybe half of it is NN and half of it is treatment. I can’t tell … percentage-wise. But if you understand, I think the whole thing has been … it has probably been extremely important. Because she has taken her time and so on and it was fantastic.”* (Participant no. 11).

## Discussion

This study aimed to explore patient experiences during treatment with ESK utilizing the person-centred nursing framework [13, 22]. Patient experiences generally matched the PCC framework. *Prerequisites* incorporated meeting competent, engaged nurses and being treated with respect; the *care environment* allowing flexibility of a supportive organization that was able to adapt to patients’ individual needs, and that patients were comfortable and had a private space; the *person-centred process* embodied that patients felt supported, valued as individuals and involved in their own care; and *expected outcomes* equated to feeling a sense of security, ultimately affecting treatment success.

### Prerequisites

The findings demonstrate that patients perceived nurses at the psychiatric clinic as competent in psychiatric treatments and experienced in dealing with ESK. Research shows that meeting competent nurses who demonstrate caring abilities is fundamental to building patients’ trust [22], which in turn is a key factor in achieving positive health outcomes [23]. Nurses with knowledge and experience in treating individuals with mental health issues tend to have less stigmatized attitudes, which promotes a good patient-provider relationship [24]. The quality of the relationship between the nurse and the patient mainly depends on the nurse’s skills, such as self-awareness, communication ability, and the ability to show empathy [25]. Nurses must be skilled in communicating with patients as unique individuals, employ active listening, and ask questions [26]. Insufficient information about medication, inaccessible care, an unprofessional attitude, and a lack of professional counselling can be barriers to recovery from TRD [27]. This entails that nurses involved in treatment with ESK must have relevant knowledge of all aspects of the medication; pharmacokinetics, pharmacodynamics, potential side effects and expected treatment effect, to instil trust and promote recovery.

Patients in the current study also felt that nurses were engaged and had a genuine desire for their well-being, which made them feel more comfortable dealing with uncomfortable experiences associated with ESK. In PCC, nurses’ competence in communication and the ability to build relationships and recognize patients’ unique needs is essential to providing safe and secure care. It is also important for nurses to be diligent in their work and have a desire to provide the best care for the patient [14]. In the current study, patients identified that soft, friendly, kind, and calm personal qualities of nurses were associated with receiving good treatment. This conforms with the research on showing patients respect and providing good treatment, and the multifactorial nature of recovery according to patients’ beliefs [28]. Respectful treatment from nurses may be based on personal attributes such as caring, empathy, insightfulness, respectfulness, and competence [29]. However, personal attributes are also closely linked to organizational factors, such as support and flexibility, ultimately providing favourable conditions for good nursing care. The organization, or care environment, according to the PCC nursing framework, also needs to provide an opportunity for nurses to develop and safeguard their competence.

The importance of the physical care environment, also a part of the PCC construct, was stressed during treatment with ESK. Several patients appreciated that the clinic had a home-like environment, rather than a traditional hospital setting. An environment that instils calm and comfort promotes the patient’s well-being and health [13]. Patients wanted to be able to sit or lie comfortably while under the influence of ESK. It was important that the room was quiet and that they had the opportunity to sit alone, but using the same room for every treatment was not emphasized as necessary. This entails that clinics using ESK should enable patients to have access to private and quiet rooms with comfortable seating. The patients’ preferences for a private space need to be balanced with clinical supervision requirements for the safety of the patient during treatment. The rooms should be easily accessible for the nurses, with blood-pressure apparatus for monitoring possible hypertension and other side-effects such as vomiting and loss of consciousness. The rooms should also provide means for the patient to call for attention. The need for a private room might limit treatment of multiple patients simultaneously due to restrictions of space.

Some patients in the current study appreciated the flexibility in terms of arranging appointment times. According to Brand and Pollock [30], being flexible in the organization means adapting to, and meeting the patients’ specific needs, which also inspires trust. Thus, a supportive and flexible organization is essential as routines, power structures, attitudes, physical environment, and standardized procedures may be factors that otherwise limit the implementation of PCC [31]. Other studies show that time constraints and policies prevent nurses from interacting with patients in a meaningful way, and the focus is on the patient’s diagnosis rather than a holistic view of the patient and the care surrounding the patient. With no chance to take part in the patient’s experiences, values, and opinions, individual care and participation become unattainable [32–34]. Developing a culture of care that values the relationship between nurses and patients can improve the quality of care and promote patient recovery from depression [28].

In the current study, patients noted that the ways in which nurses relate to, and treat patients, plays a significant role in patients’ well-being, and how patients perceive their treatment’s effectiveness. This is particularly true in PCC, where building a good relationship between nurses and patients is crucial. According to a study by Chambers et al. [35] and DeCou & Vidair [36], nurses must be non-judgemental, open-minded, good listeners, nice, calm, and accepting, and treat patients as whole humans and not cases. A good relationship is built when nurses commit to their patients and consider them to be equal partners in the decision-making process. In the current study, during the monitoring period, patients had varying preferences regarding frequency of supervision, and room environment, such as sound and light. Most patients felt that they had a say in how they wanted it and that the nurses adapted accordingly. This flexibility in supervision entails that nurses must have time assigned for each patient receiving treatment, as supervision will be more frequent at times and less frequent at other times. A few patients felt that they were unable to influence their care in some respects, such as wanting a dose or interval of treatment or wanting treatment at home, which went against the guidelines for the administration of ESK. Ensuring the patient understands the rationale behind guidelines, particularly from a safety perspective, is important for the relationship and ultimately, the treatment outcome. According to PCC, participation is a crucial aspect and involving the patients, and adapting to their needs, is essential for high quality in person-centred processes, and ultimately to achieving desired treatment outcomes. In the current study, patients described participation as being involved in decision-making processes regarding their care, where they were asked what they thought was best for them, and the nurse accommodated their preferences. They felt respected and valued when they could participate in their care, and that their opinions mattered. When patients are involved in their own care, they trust nurses more and are more likely to comply with treatment [28]. According to Sheldon [37], the ability of nurses to listen to the patient is an important aspect of involving the patient in decisions. Barriers to participation may arise when carers have a poor attitude, do not take the patient seriously, or are short of time [27]. In the current study, several patients highlighted the importance of having time to talk, not only about their illness, but about life in general. They appreciated it when nurses took the time to listen and made them feel seen as individuals. This is supported by Ekman et al. [11], who argue that the opportunity to talk about one’s well-being, life, and experiences is the basis for a good relationship between patients and nurses. It is likely that the substantial and recurring time spent together by nurse and patient during ESK contributes to high-quality, person-centred processes. Thus, ESK treatment and administration must be regarded as an encounter allowing interaction between patient and nurse, and not a quick and mechanical handing over of medication. This is also in line with the PCC processes, which emphasize the importance of the relationship between patient and nurse in establishing trust.

Patients stated that feeling secure during their treatment with ESK is critical, as the medication can cause unpleasant side effects. Thus, it is essential for patients to feel secure in their environment and with the nurse. Patients identified several factors that contributed to their sense of security, such as a comfortable and welcoming environment, seeing the same nurse at each visit, receiving care from professional and empathetic nurses, and having a say in their treatment. This aligns with Mollon’s [38] description of security as an emotional state that is impacted by memories and experiences. A peaceful and inviting care environment is crucial for patients’ sense of security, as both the physical and social environment impact their sense of security [39]. Building a robust relationship with a nurse involves continuously meeting the same nurse, which fosters trust and confidence, as long as the nurse is competent, responsive, and skilled in communication [40]. Continuity of care, such as seeing the same nurse at each visit, was perceived as fostering a sense of security and enabling individualized interactions. When the nurse knows the patient, it’s easier to pick up on non-verbal communication, such as mood, and facial expressions, and be able to identify any concerns and fears, thereby resulting in more effective care for patients [41, 42]. Today’s healthcare organizations struggle with providing continuous contact with the same healthcare professionals for patients, which potentially prolongs the effective recovery of TRD [43]. Clinics using ESK should prioritize staff continuity and strive to ensure that patients meet the same nurse to enhance the recovery process.

Patients in the current study reported positive outcomes from ESK, including reduced anxiety and improved ability to cope with daily activities, which helped them avoid negative thoughts and feelings. It is worth noting that some patients were uncertain whether their increased well-being was due to the medication per se or the nursing care and interactions with nurses. This underscores the importance of a person-centred approach to patients’ recovery.

### Methodological considerations

As this study aims to describe patients’ experiences, a qualitative approach is advantageous, because it provides a nuanced picture of their experiences. The quality of qualitative studies is reviewed based on credibility, dependability, transferability, and confirmability [44]. A detailed description has been made based on the selection and analysis to enhance *credibility*. Twenty interviews were deemed sufficient for the purpose of the study, to discover similarities and variations in patients’ experiences. One limitation may be that most interviews were conducted digitally or over the phone, meaning that facial expressions and body language were missing. However, there are advantages to conducting interviews digitally, as it can be easier to share private information when there is a sense of anonymity and distance from the interviewer. Although two members of the research team had considerable experience as psychiatric nurses, the interviewer, LK, who conducted most of the interviews, had no prior experience of treating patients with ESK, nor any personal knowledge of the out-patient clinic – which enhances credibility. This study used an interview guide to ensure consistency in the questions asked and encourage participants to share their experiences freely, thus increasing *dependability* [45].

Diversity in terms of gender, age, and illness duration in this study encourages a breadth of experiences, which strengthens *transferability* [46, 47]. The study was conducted in a single clinic, which can affect transferability. Interviewing patients from multiple clinics with diverse environments, policies, and nurses would increase transferability [44]. Most patients responded well to treatment and had positive experiences with the care they received, expressing gratitude for the opportunity to try the treatment method. Therefore, it can be questioned whether critical perspectives, such as poor treatment outcomes or negative experiences, are underrepresented, and perhaps those who chose not to participate in the study opted out for this reason. TRD is a difficult condition to evaluate, as aspects such as compliance, other life events, the experience of previously failed treatments, the mood at interviews, etc., affect the patient’s experience of treatment and its effectiveness. Quotations are included in the Results section, to strengthen *confirmability* [46, 47].

To ensure trustworthiness, the study is reported in accordance with the Consolidated Criteria for Reporting Qualitative Research 32-item checklist [45].

## Conclusion

This study highlights how patients experience treatment with ESK and how this aligns with the PCC nursing framework. The results indicate that nurse competence and commitment, in conjunction with a supportive and flexible organization, contribute to patients’ well-being. Besides inter-personal skills and attributes of the nurses, patients also highlighted the importance of a supportive care environment, comfortable seating arrangements, a private space and personalized care. In addition, patients valued being recognized as unique individuals, feeling supported throughout their treatment, and being involved in decisions related to their care. Ultimately, the expected outcomes of ESK treatment, in alignment with PCC, were to achieve the desired effect of the treatment and to feel a sense of security. Patients explicitly stated that they were unable to determine whether the positive outcomes they experienced related to the effect of the medication or the influence of the nursing care.

This study emphasizes the positive impact of a PCC approach on the well-being of patients undergoing treatment with ESK. The information may improve patient experience and well-being during nasal esketamine treatment in the future, as the results of this study can serve as a recommendation for improving the quality of care in treating patients with ESK. As PCC has numerous benefits for individuals and organisations, future research should focus on identifying the factors and barriers that prevent organisations from working with a person-centred approach.

## Electronic supplementary material

Below is the link to the electronic supplementary material.

Supplementary Material 1

## Data Availability

Empirical material generated and/or analysed during the current study is not publicly available due to ethical restrictions, but is available from the corresponding author on reasonable request.

## Abbreviations

ESK: Nasal esketamine
PCC: Person-centred nursing framework
TRD: Treatment-resistant depression
