# Supplementary material for: Person-centred care during treatment with nasal esketamine — a qualitative study
Source: BMC Nurs. 2025 Mar 14;24:287. doi: 10.1186/s12912-025-02943-y (PMC11907890; doi:10.1186/s12912-025-02943-y)
Supplement: Supplementary file 1 — Supplementary Material 1 [file 12912_2025_2943_MOESM1_ESM.docx]

Interview guide EUPPNES

Person-Centered Processes:

Describe a typical treatment session?

How would you describe your interactions and communication with the healthcare staff, including doctors and nurses, during the treatment session?

In what way has your treatment been tailored to meet your individual needs and preferences?

Can you describe how decisions about your treatment are made and how you are involved in that process?

Care Environment:

How would you describe your experience of the clinic’s atmosphere and environment during the treatment?

In what way does the environment affect you?

How does the care environment impact your overall experience?

Prerequisites:

Can you tell me how your previous experiences with depression treatment have influenced your attitude and view of care and your current treatment?

What factors do you consider important for achieving successful treatment of depression?

Person-Centered Outcomes:

What results or goals do you hope to achieve with your treatment?

How do you assess if your treatment is effective and working?

What specific results/symptoms/function are most important to you?
